# Is the Proust phenomenon fact or fiction? A systematic review and meta-analysis of odor-evoked autobiographical memory

**DOI:** 10.3758/s13423-026-03025-z

**Published:** 2026-10-26

**Authors:** Ovidiu I. Bobei, Dragos Cîrneci, Ioana R. Podina

**Affiliations:** 1 Laboratory of Cognitive Clinical Sciences, University of Bucharest, Bucharest, Romania; 2 Interdisciplinary School of Doctoral Studies, University of Bucharest, Bucharest, Romania; 3 MINDCARE FOR ALL Association, Bucharest, Romania; 4 Faculty of Psychology and Education Sciences, Spiru Haret University, Bucharest, Romania; 5 COGLAB R&D Solutions, Bucharest, Romania

**Keywords:** Autobiographical memory, Odor exposure, Olfactory cues, Proust phenomenon

## Abstract

Odors are widely believed to be the most powerful trigger for autobiographical memory (AM). This meta-analysis provides the first comprehensive meta-analytic test of this claim, known as the Proust phenomenon. Eighteen experimental/quasi-experimental studies were included in this meta-analysis. Olfactory cueing was compared with a variety of cueing modalities, and seven key characteristics of evoked AMs (specificity, vividness, emotional intensity, pleasantness, rarity, evocativeness, and age of AMs) were assessed. Olfactory cues showed a greater effect (advantage) over other cue types for emotional intensity, pleasantness, evocativeness, and age of AMs. Sample size was negatively associated with effect sizes for emotional intensity, pleasantness, and rarity, although the overall effect for rarity was not significant. Higher proportions of females and more recent publication years were each associated with smaller effects for evocativeness and pleasantness, respectively. Subgroup analyses showed that the advantage of olfactory cues for emotional intensity was stronger in within-subject than between-subject designs. Moreover, their advantage in terms of pleasantness was greatest relative to label cues, followed by visual and auditory cues. Although olfactory cues showed an advantage for four characteristics of AMs, these results do not fully confirm the Proust phenomenon. Overall, these findings suggest that the olfactory advantage is selective rather than universal, and future studies should clarify the conditions under which it emerges.

## Introduction

Sensory cueing is one of the most widely used methods for evoking autobiographical memories (AMs) (Fernández-Pérez et al., 2022). Studies have shown their potential as a therapeutic tool (Hanaoka et al., 2018; Leret & Visch, 2017) with promising effects in pathologies such as depression (Canbeyli, 2022), cognitive decline (Sunami et al., 2025), and dementia (D’Andrea et al., 2022; Pinto et al., 2020).

Although there are five senses through which sensory cueing can occur – olfactory, auditory, visual, gustatory, and tactile (Ernst et al., 2021) – olfactory cues have received particular attention due to the characteristics of the AMs they evoke, commonly referred to as the Proust phenomenon. In the original passage, Proust describes a flavor experience that spontaneously triggers a forgotten childhood memory. As Jellinek (2004) noted, this experience involved not only smell but also taste, texture, and temperature cues, making the term “Proust phenomenon” a conceptual simplification when applied exclusively to olfaction. Nevertheless, this episode has inspired a growing body of experimental studies and reviews aimed at identifying and testing the characteristics of odor-evoked AMs, and the term has been retained in the literature as a label for odor-evoked AMs.

Early work (Chu & Downes, 2000) characterized odor-evoked AMs by three key features: *vividness,* i.e., how vivid/clear the event is in the mind (Chu & Downes, 2002); *emotional intensity,* i.e., with intense affective tone (Herz & Cupchik, 1995); and *age of AMs,* i.e., the AMs’ tendency to be very old or more likely to come from childhood (Chu & Downes, 2000). Beyond these features, research has examined additional characteristics associated with the Proust phenomenon, including *specificity,* i.e., how detailed the evoked AM is, regarding the place and time (Herz & Schooler, 2002); *evocativeness* (sense of being brought back in time), i.e., the intensity with which the evoked AM is relived (Herz & Schooler, 2002); and *rarity*, i.e., the frequency with which an AM is thought of (Rubin et al., 1984). Most of them were synthesized in the LOVER acronym (i.e., Limbic, Old, Vivid, Emotional, and Rare) (Larsson et al., 2014), which has been widely used to define odor-evoked AMs (Hackländer et al., 2019).

Related to emotional intensity captured within this framework, *pleasantness* (i.e., the pleasure/positive valence of the AMs) has also been analyzed as a feature of odor-evoked AMs. Although not explicitly recognized as a characteristic associated with the Proust phenomenon (Chu & Downes, 2000; Hackländer et al., 2019), studies have included this dimension, in part because odor-evoked AMs are often reported as more pleasant than those triggered by other sensory cues (Arshamian et al., 2013; Miles & Berntsen, 2011). Such findings align with the therapeutic potential of olfactory cueing, given evidence that odors evoking positive AMs can beneficially influence emotional states and stress responses (Herz, 2016).

A key hypothesis proposed to explain these features concerns the unique neuroanatomical organization of the olfactory system (De Beukelaer et al., 2023; Kontaris et al., 2020; Saive et al., 2014). Specifically, unlike other senses, olfaction has direct connections to the amygdala and hippocampus, two structures central to emotional processing and memory, via the olfactory bulb and primary olfactory cortex (Saive et al., 2014; Soudry et al., 2011).

Despite this neuroanatomical hypothesis, experimental studies that have examined the advantage of olfactory cueing (i.e., the ability to evoke more emotional, pleasant, evocative, vivid, specific, rare, and old AMs) in contrast to other cueing modalities (e.g., visual, auditory) have yielded mixed results (e.g., de Bruijn & Bender, 2018; Herz & Schooler, 2002; Miles & Berntsen, 2011; Yar et al., 2025), and meta-analytic evidence remains limited. To date, only one review has provided more than narrative findings (Hackländer et al., 2019). The analyses performed in this review revealed that odor-evoked AMs were indeed more emotionally intense, pleasant, vivid, rare, old, and generally accompanied by a stronger sense of being brought back in time (evocativeness) than AMs evoked by other cues. Nevertheless, several methodological concerns mentioned by the authors are worth noting. Firstly, some effect sizes in this review may have been misestimated. For example, regarding memory vividness, the authors assigned a negative effect size in their analysis when no numerical data or figures were available. Secondly, some data extracted for the analyses were preferentially selected, such as including only childhood-odor data in the vividness analysis (Hackländer et al., 2019), which may have led to an overestimation of the effect.

In addition to the authors’ arguments, several important limitations are worth discussing. Firstly, some of the effect sizes calculated in this review (Hackländer et al., 2019) were based on data not reported in the included studies. For example, where effects were not significant, the authors interpreted this by including an effect size of 0. Secondly, this review also did not consider outcomes analyzed in all included studies, such as specificity. Finally, no analyses were conducted to assess potential moderators, study quality, or publication bias.

## The current meta-analysis

In light of these limitations, the present study is, to our knowledge, the first meta-analysis to test the empirical assumptions of the Proust phenomenon, i.e., the relative advantage of olfactory cues over other cue types in evoking more vivid, emotionally intense, evocative, specific, rare, and older AMs, as well as more pleasant AMs, with pleasantness being a dimension frequently examined in the literature as a characteristic of odor-evoked AMs.

Furthermore, to ensure a rigorous analysis of the comparative advantage of olfactory cues over other cues in AM retrieval, the present meta-analysis examines several moderators that may explain variability in the effect size estimates. These moderators are divided into: *study characteristics* (e.g., year of publication, study design); *sample characteristics* (e.g., mean age, sample size, gender); and *procedure* (e.g., cue method, cue type).

Study characteristics, such as publication year or study design, are often analyzed as potential moderators in meta-analyses (e.g., Li et al., 2026; Podina et al., 2023). For instance, study design can influence effect sizes. Compared with between-subject designs, within-subject designs tend to yield higher statistical power, as each participant is exposed to all conditions (Charness et al., 2012; Montoya, 2023).

Other important moderators are related to the sample characteristics. Age, for example, plays a well-documented role in olfactory perception. Some studies show that performance in odor perception and identification declines with age (Doty & Kamath, 2014). Additionally, there are gender differences in olfactory perception. A recent meta-analysis examining gender differences in olfactory perception found a superiority of women over men in terms of olfactory identification, olfactory threshold, and olfactory discrimination, highlighting the need to control for gender differences in future studies (Sorokowski et al., 2019).

Similarly, procedure-related variables can serve as potential moderators. For example, the cue method has been proposed in previous research as a possible source of variability (Hackländer et al., 2019). The cue method refers to the procedure for presenting sensory cues. In the single-cue method, each assigned participant first receives the cue (e.g., a smell or sound) and then describes the AM evoked by that cue. In the double-cue method, by contrast, each participant is first presented with a label (either verbal or displayed on a screen) and asked to describe the evoked AM. Afterward, the participant is assigned to a sensory condition (e.g., olfactory, auditory, or the same label) and, based on the cue in that condition, is asked to describe the AM again (de Bruijn & Bender, 2018; Hackländer et al., 2019). However, because no moderator analyses have been conducted, previous research has been unable to determine whether this factor systematically explains differences in AM outcomes.

Moreover, the cue type may have a moderating effect on AM outcomes. The cue type refers to which alternative cue (e.g., auditory, visual) is being compared to olfaction. Given that different sensory cues vary in their ability to evoke AMs (Goddard et al., 2005), the effect size estimates reported across studies may partly reflect characteristics of the specific alternative compared, rather than a genuine olfactory advantage.

To summarize, this meta-analysis aimed to examine the relative advantage of olfactory cues over other cue types in evoking more vivid, emotionally intense, pleasant, evocative, specific, rare, and older AMs. Moreover, it aimed to investigate possible moderators that may explain the variability in effect sizes across studies. Finally, to assess the quality of the included studies, the current work aimed to evaluate their risk of bias and analyze publication bias.

## Methods

A systematic search of PsycInfo, PubMed, Web of Science, and Scopus was conducted from June through September 2024 and updated in October 2025 to identify potentially relevant studies. We also screened the reference lists of the most recent reviews on odor-evoked AMs (Gisquet-Verrier & Riccio, 2024; Hackländer et al., 2019), and conducted complementary searches in Google Scholar to ensure comprehensive coverage of the literature. The search terms used were: (olfactory stimulation OR odor OR olfaction OR olfactory cues OR odor exposure OR olfactory priming OR olfactory processing OR olfactory perception OR smell OR olfact* OR sensory stimulation) AND (autobiographical memory OR evoked autobiographical memories OR autobiographical recall OR autobiographical memory retrieval OR autobiographical memory activation OR enhancement of autobiographical memory OR specific autobiographical memory OR episodic memories).

### Inclusion/exclusion criteria

Studies were included if they: (a) used a method of evoking AMs through olfactory cueing; (b) were experimental studies (i.e., involving manipulation of cueing with random assignment to conditions) or quasi-experimental studies (i.e., involving manipulation of cueing without random assignment to conditions), using either a within-subject or a between-subject design; (c) measured at least one of the characteristics of evoked AMs (specificity, vividness, evocativeness, emotional intensity, pleasantness, rarity, or age of AMs); and (d) provided enough quantitative data to compute an effect size. To calculate effect size, included studies were required to report means, standard deviations, and sample size. Alternatively, where these data were not available, Cohen’s *d* or F statistics reflecting the contrast between olfactory and other cue types could be extracted.

Studies were excluded if they: (a) used AM training interventions; (b) were case studies or qualitative and narrative studies; (c) analyzed recollections other than personal life events (e.g., episodic memory). The episodic memory paradigm differs from the autobiographical one in several aspects. Firstly, one characteristic of odor-evoked AMs is their childhood age. In the episodic paradigm, where memories are created artificially in the laboratory, this variable cannot be tested, as the encoding-to-recall interval is generally a few minutes. The same limitation could also apply to AM rarity. A memory that has existed for only a few minutes cannot yet have accumulated sufficient history to be frequently or rarely retrieved. Thus, the included studies should use a methodology that allows testing of any variable of odor-evoked AMs. Furthermore, in the episodic paradigm, the event is predetermined by the investigators, which does not allow for the recall of a real-life event in response to a sensory cue (Chu & Downes, 2000). However, given that some studies treat episodic and autobiographical memory similarly (Gardiner, 2001; Kopelman & Kapur, 2001), we included episodic memory as a search term to ensure that potentially relevant studies were not excluded.

Additionally, only English-language publications were analyzed. There were no restrictions regarding publication year, geographical area, or unpublished manuscripts.

### Data extraction

**Fig. 1:**
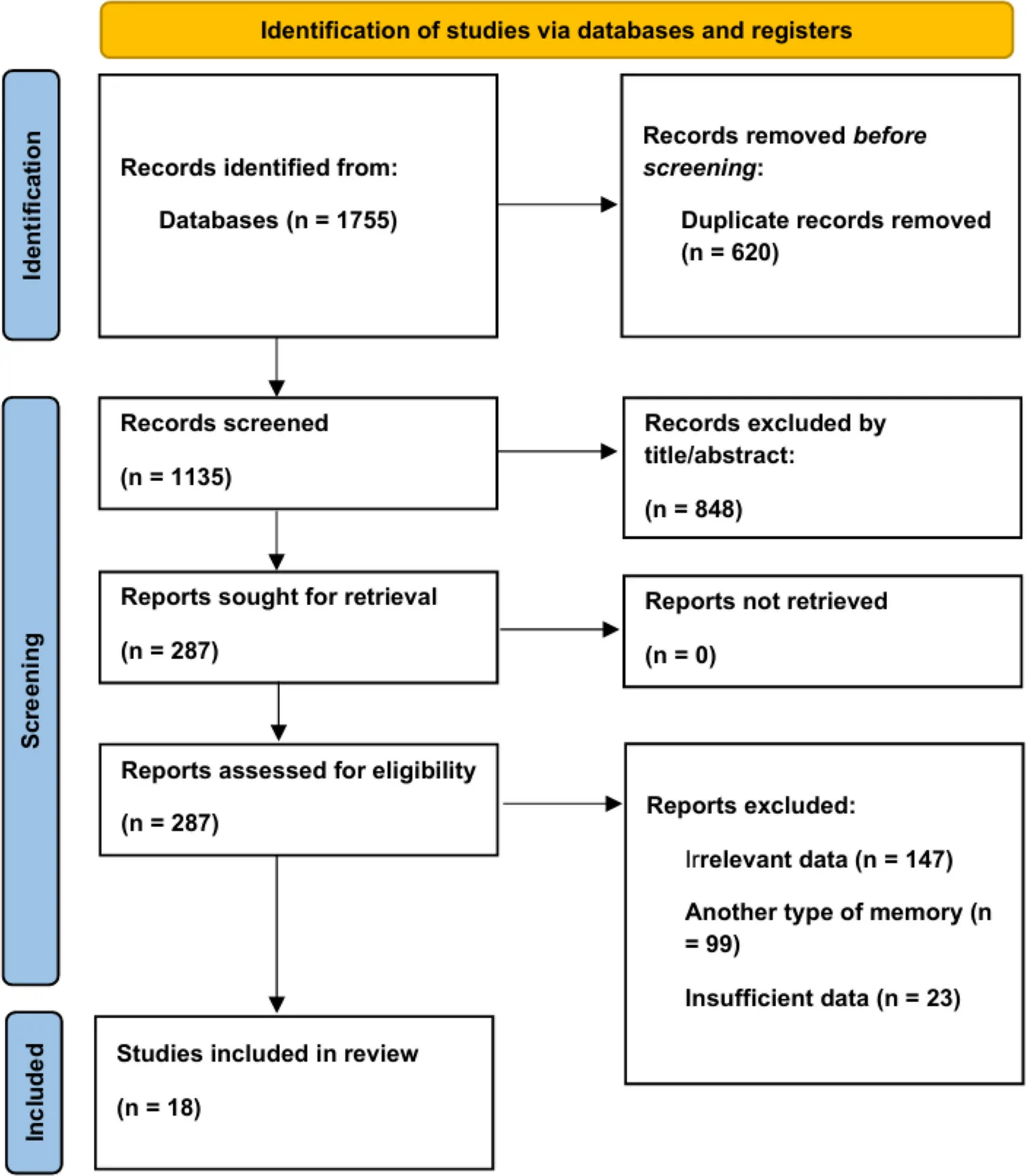
PRISMA flow diagram of the studies selection process

Duplicate studies were removed after a comprehensive search, and the remaining titles and abstracts were examined, with ineligible papers being excluded. The full texts were then retrieved and vetted for inclusion. Figure 1 describes the study selection process. Eighteen full-text papers met the inclusion/exclusion criteria and were included in the meta-analysis. Each study included in the analyses reported data for at least one contrast between olfactory and another cue type (e.g., olfactory vs. visual), as well as one outcome: *specificity, vividness, emotional intensity, pleasantness, rarity, evocativeness,* or *age of AMs*.

The following data were extracted from each study: (a) *study characteristics*, i.e., authors’ names, publication year, study design (i.e., within-subject or between-subject design), cue types (e.g., auditory, visual), cue method (i.e., single or double cue); (b) *sample characteristics*: sample size, mean age, percentage of females, with or without clinical diagnosis; (c) *AM characteristics,* i.e., data regarding specificity, vividness, emotional intensity, pleasantness, rarity, evocativeness, or age of AMs; (d) *other relevant characteristics* for moderator analyses (i.e., duration of exposure to stimuli, number of stimuli used).

### Outcomes and moderators

The outcomes analyzed were: *specificity*, *vividness, sense of being brought back in time/evocativeness*, *emotional intensity, pleasantness, rarity,* and *age of AMs.* These variables represent the most frequently examined dimensions of odor-evoked AMs in the literature (Chu & Downes, 2000; de Bruijn & Bender, 2018; Hackländer et al., 2019; Larsson et al., 2014).

We also considered a comprehensive list of potential moderators. These moderators were divided into: *characteristics of the included studies* (i.e., publication year and study design); *characteristics related to the sample* (i.e., mean age, sample size, percentage of females); and *procedure* (i.e., cue type, cue method, duration of exposure to stimuli, and number of stimuli used). Publication year, sample size, mean age, percentage of females, duration of exposure to stimuli (measured in seconds), and number of stimuli used were coded as continuous moderators. Cue type, study design, and cue method were treated as categorical moderators. Each continuous moderator was entered in its original units (e.g., mean age in years). *Cue type* was divided into the following categories: auditory cues, visual cues, and label cues (either as a verbal label or represented by the display of the cue word on a screen). *Study design* was divided into two categories: within-subjects design and between-subjects design. *Cue method* was divided into the following categories: single-cue, double-cue, or mixed (where the methods were combined).

### Quality assessment

To estimate the quality of the included studies, three evaluation criteria of the Joanna Briggs Institute (JBI) critical appraisal tool were used as follows: (a) selection bias, (b) detection bias, and (c) statistical analysis bias. The JBI critical appraisal tool is one of the standards for evaluating the risk of bias alongside the Cochrane Collaboration Risk of Bias Tool (Higgins et al., 2011), being ideal for assessing the quality of experimental studies, providing a comprehensive assessment (Barker et al., 2023).

**Fig. 2:**
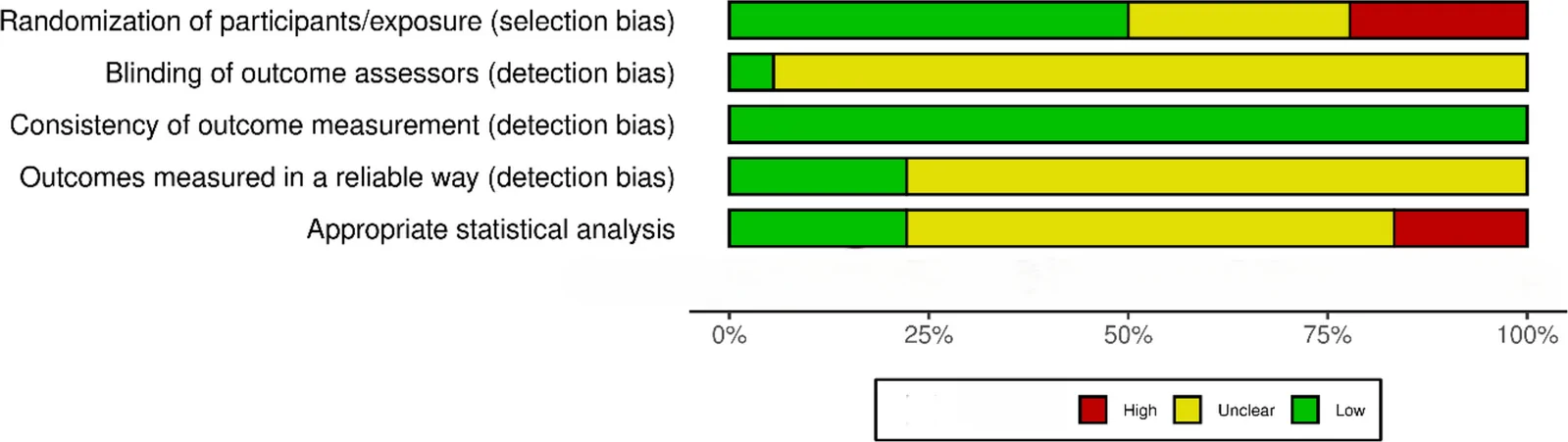
Risk-of-bias graph: Review of authors’ judgments about each risk of bias item presented as percentages across all included studies

The JBI was manually applied to each study included in the meta-analysis, with scores assigned as “yes” or low risk of bias (green), “no” or high risk of bias (red), and “some concerns” (yellow). For each study, the total number of “yes” responses was calculated based on the five questions, making up the three criteria mentioned above. For this evaluation, questions that are not applicable to the type of study design were excluded (e.g., questions referring to the follow-up period of the participants were excluded). The items used are described in detail below, and the graph illustrating the findings can be found in the *Results* section (Fig. 2).

(1) “Was true randomization used for assignment of participants to experimental groups?” (2) “Were outcomes assessors blind to treatment assignment?” (3) “Were outcomes measured in the same way for experimental groups?” (4) “Were outcomes measured in a reliable way?” (5) “Was appropriate statistical analysis used?”.

### Meta-analytic procedure

The study followed the Preferred Reporting Items for Systematic Reviews and Meta-Analyses (PRISMA) guidelines, and the Comprehensive Meta-Analysis Software (CMA) version 3 was used to calculate effect sizes. Effect sizes were calculated using Hedges’ *g*, which adjusts for bias in small samples (Hedges & Vevea, 1998), and were computed from means, standard deviations, and sample size; where these were not reported, Cohen’s *d* or F statistics were extracted instead (Cohen, 2013). Similar to Cohen’s *d*, Hedges’ *g* is interpreted as follows: 0.2–0.5 indicates a small effect, 0.5–0.8 a medium effect, and 0.8 or greater a large effect (Cohen, 2013). A positive Hedges’ *g* value indicated an advantage of olfactory cues relative to other cue types.

Separate meta-analyses were conducted for each of the seven AM outcomes (e.g., vividness, evocativeness), each including all available contrasts between olfactory cues and other cue types for that outcome. Where multiple contrasts were reported within the same study (e.g., olfactory vs. visual and olfactory vs. auditory cues), these were treated as study subgroups to enable direct contrast between olfactory and each cue type. To address the dependency introduced by multiple contrasts sharing the same olfactory group, we applied a sample-splitting approach. Specifically, the olfactory sample size was evenly divided across contrasts. This method, recommended for multi-arm trials, helps to prevent double counting of participants and reduces potential bias in meta-analyses (Axon et al., 2023).

### Overall effect sizes

To estimate the pooled effect sizes of olfactory cues relative to other cue types on AM outcomes, a random-effects model was used, with a random effect to account for between-study variability in true effect sizes (Borenstein et al., 2010; Hedges & Vevea, 1998). In this model, no moderator variable was included; it estimated an overall pooled effect size for each AM outcome, representing the average advantage of olfactory cues over other cue types across all included studies. Heterogeneity was measured using the *I*^2^ statistic and was interpreted as 0% for no observed heterogeneity, 25% for low heterogeneity, 50% for moderate heterogeneity, and 75% or more for high heterogeneity. Low heterogeneity indicates consistency in effect sizes across studies, strengthening confidence in the pooled effect size. Conversely, moderate-to-high heterogeneity signals that study-level characteristics may systematically influence outcomes, which moderator analyses can help explain.

### Moderator analyses

Moderator analyses used the same random-effects model, with one addition: a single moderator variable (categorical or continuous) was included to examine whether it accounted for variability in the effect size. Rather than estimating a single pooled effect across all studies, as above, this model estimated how the effect size varied according to the moderator. Categorical moderators were analyzed by subgroup analyses using mixed-effects models where a random effects model is used to combine studies within each subgroup. Continuous moderators were tested in a separate univariate meta-regression model using a Z-distribution approach (Borenstein et al., 2009), in which each continuous moderator served as a predictor for the effect size. Each moderator was tested in a separate model rather than simultaneously in a single multivariate model, because the relatively small number of included studies limited statistical power for multivariate approaches.

### Publication bias

Publication bias was assessed using a three-step approach. First, the funnel plot asymmetry was inspected visually. The funnel plot provides a graphical representation of the distribution of effect sizes relative to their standard errors. A substantial asymmetry in the graphical representation may indicate a volume of unpublished studies. Second, Egger’s regression test was used to assess funnel plot asymmetry, with a significant result (*p* <.05) indicating potential publication bias (Egger et al., 1997). Third, Duval and Tweedie’s trim-and-fill procedure was applied to estimate the number of potential omitted studies and to adjust the effect size accordingly (Duval & Tweedie, 2000). Together, these three methods provide converging evidence regarding the likelihood and potential impact of publication bias on the pooled effect size.

### Sensitivity analysis

Finally, to control for potential effects that may be introduced by the inclusion of studies with clinical populations (El Haj et al., 2018; Leiker et al., 2024; Lopis et al., 2021) where autobiographical memory may be impaired (Irish, 2023; Weiss-Cowie et al., 2023), a sensitivity analysis was performed in which values from these samples were excluded to ensure that the overall effect remained similar.

This meta-analysis has been pre-registered in accordance with good research practice guidelines. Pre-registration is available on PROSPERO International Prospective Register of Systematic Reviews (registration number: CRD42025630034).

## Results

### Selection and inclusion of studies

The database search returned 1,755 records, of which 1,135 remained after eliminating duplicates. We excluded 848 records based on title/abstract review and retrieved the full text of 287 articles. Figure 1 illustrates the flowchart of the selection and inclusion processes, following the PRISMA guidelines (Moher et al., 2009). A total of 18 studies met our eligibility criteria and were included in the meta-analysis.

### Characteristics of included studies

The characteristics of the included studies are presented in Table 1. The 18 included studies yielded 24 comparisons between olfactory cues and other cue types for emotional intensity, 19 for pleasantness, 18 for evocativeness and vividness, 12 for specificity, 11 for rarity, and eight for age of AMs. Also, the included studies allowed 42 *olfactory-visual* comparisons, 26 *olfactory-auditory* comparisons, and 42 *olfactory-label* comparisons *(*either a verbal label or represented by the display of the cue word on a screen). Finally, the 18 studies analyzed included a total of 1,330 participants (*M* = 40.71 years; *SD* = 24.56).

**Table 1:** Characteristics of the studies included in the meta-analysis

Study	Mean age, y; % female	Sample size	Cue type compared	Outcomes	Design type	Clinical population	Cue method	Odor presented	Rating scales
Arshamian et al. (2013)	24.7 53%	15	Label	E; EV; P; R*	Within	No	Single	Opaque glass bottles	Likert
Chu and Downes (2002) Exp. 1	-	42	Label	V; P	Between	No	Double	Wrapped glass jar	Likert
Cox (2020)	20.47 78%	108	Auditory; Label	E; EV; V; P; S; Age M	Between	No	Single & Double	Sealed containers	Likert
de Bruijn and Bender (2018)	20 79%	170	Visual	V; S; E	Between	No	Double	Metal cylindrical containers	Likert
El Haj et al. (2018)	71–73 72%	58	Auditory	P; EV; S	Within for cue type	No and Yes	Single	Small bottle of essential oils	Likert
Goddard et al. (2005)	24.54 50%	48	Visual; Label	Age M; V*	Within for cue type	No	Single	Covered jar	Likert
Herz (2004)	33 60%	70	Label (selection phase); Auditory; Visual	S; V; E; EV	Within	No	Double	Sealed containers	Likert
Herz and Schooler (2002)	33.8 56%	45	Label (selection phase); Visual	S; V; E; EV	Between	No	Double	Opaque plastic bottle	Likert
Herz et al. (2004)	22 100%	5	Visual	E	Within	No	Single	Olfactometer	Likert
Leiker et al. (2024)	30 81%	32	Label	V; E; R	Within	Yes	Single	Small opaque glass jars	Likert
Lopis et al. (2021)	80.9; 80.1; 22.2 65%; 75%; 63%	180	Visual; Auditory*	V*; E*; R	Between	No and Yes	Single	Sniffing sticks	Likert
Miles and Berntsen (2011)	22.98 82%	89	Visual; Label	E; P; EV; R; Age M	Between	No	Single	Opaque jars	Likert
Rubin et al. (1984) Exp. 1	-	40	Label	P; R; V*; Age M*	Between	No	Single	Opaque glass bottle	Likert
van Hoogeveen (2020)	20.8 83%	102	Visual	S; V; E*; EV; P	Between	No	Single & Double	Metal container	Likert
Willander and Larsson (2006)	74.32 57%	93	Visual; Label	E; EV; R; V*; S*; P* Age M*	Between	No	Single	Black glass jars	Likert & SAM
Willander and Larsson (2007)	71.69 69%	72	Label	P; E; EV; V*; Age M*	Between	No	Single	Non-translucent glass jars	Likert
Willander et al. (2015)	23.94 74%	62	Visual; Auditory	P; E; EV; V; Age M*	Between	No	Single	Non-translucent glass jars	Likert
Yar et al. (2025)	22.97 59%	99	Visual; Label	V; P; E; R	Between	No and Subclinical	Single	Non-translucent glass jar	Likert & VAS

E = emotional intensity; EV = evocativeness; P = pleasantness; V = vividness; R = rarity; S = specificity; Age M = age of autobiographical memories

*No data were reported to calculate the effect size. Only the cue types compared that are relevant to the present meta-analysis are reported

### Risk of bias

Most studies presented some concerns (yellow) for the outcomes measured in a reliable way; only four out of 18 studies had a low risk (green) for this criterion. All studies have shown a low risk (green) in terms of the consistency of outcome measurement, while 17 out of 18 studies presented some concerns regarding blinding of assessors and one low risk. Five of the 18 studies presented some concerns in the participant randomization/exposure process, while four had a high risk of bias (red) and nine a low risk. Regarding the criterion of appropriate statistical analysis, 11 out of 18 studies raised some concerns, four presented a low risk of bias, and three presented a high risk of bias (red). Figure 2 illustrates the percentage of studies with low risk of bias, some concerns (i.e., insufficient information was provided), and high risk of bias for each of the five quality criteria analyzed.

### Main results

#### Emotional intensity and pleasantness

The overall effect indicated a small, but significant, effect of olfactory cueing on pleasantness (*g* = 0.20, 95% CI [0.03; 0.37]) and emotional intensity (*g* = 0.23, 95% CI [0.07; 0.38]) compared with other cue types. The effects were maintained after excluding the clinical samples: pleasantness (*g* = 0.22, 95% CI [0.05; 0.40]) and emotional intensity (*g* = 0.21, 95% CI [0.05; 0.37]). This suggests that odor-evoked AMs are perceived as more pleasant and emotionally intense compared to AMs evoked by other cues. The heterogeneity index of the overall effect indicated a low level of heterogeneity (*I*^*2*^ = 11% for pleasantness and *I*^*2*^ = 34% for emotional intensity) (see Table 2).

**Table 2:** Overall results and subgroup analyses for emotional intensity and pleasantness of evoked autobiographical memories (olfactory cue vs. other cue types)

		*k*	*g*	95% CI	*I*^*2*^	*p*
**Emotional intensity**						
All studies		24	0.23	[0.07; 0.38]	34	**.003**
Clinical sample excluded		23	0.21	[0.05; 0.37]	34	**.008**
**Subgroup analyses**						
Study design	Within	6	0.54	[0.26; 0.82]	23	**.009**
	Between	18	0.12	[− 0.03; 0.27]	16	
Cue type*	Visual	11	0.31	[0.05; 0.57]		.62
	Auditory	4	0.09	[− 0.27; 0.46]		
	Label	9	0.22	[− 0.01; 0.46]		
Cue method	Single	17	0.20	[− 0.01; 0.42]		.68
	Double	7	0.27	[0.05; 0.48]		
**Pleasantness**						
All studies		19	0.20	[0.03; 0.37]	11	**.01**
Clinical sample excluded		18	0.22	[0.05; 0.40]	12	**.01**
**Subgroup analyses**						
Study design	Within	3	0.20	[− 0.34; 0.75]		.92
	Between	16	0.23	[0.05; 0.40]		
Cue type	Visual	5	0.33	[0.02; 0.63]	0	**.01**
	Auditory	5	− 0.15	[− 0.44; 0.12]	0	
	Label	9	0.38	[0.14; 0.61]	0	
Cue method	Single	15	0.23	[0.06; 0.41]		.35
	Double	3	− 0.15	[− 0.77; 0.45]		
	Mixed	1	0.42	[− 0.09; 0.93]		

k = number of comparisons. All results in the table are reported with Hedges *g*, using a random-effects model; a positive effect indicates the advantage of olfactory cues compared to other cue types. * Cue type refers to the alternative cues (e.g., auditory, visual) against which the olfactory cues were compared

#### Evocativeness

The overall effect size for evocativeness was small but significant (*g* = 0.22, 95% CI [0.08; 0.37]) in favor of olfactory cues compared with other cue types. The effect remained significant after excluding clinical samples (*g* = 0.24, 95% CI [0.08; 0.39]). This means that odor-evoked AMs provide a stronger sense of being brought back in time than AMs evoked by other cues. The level of heterogeneity was low (*I*^*2*^ = 9%) (see Table 3).

**Table 3:** Overall results and subgroup analyses for evocativeness of evoked autobiographical memories (olfactory cue vs. other cue types)

		*k*	*g*	95% CI	*I*^*2*^	*p*
**Evocativeness**						
All studies		18	0.22	[0.08; 0.37]	9	**.002**
Clinical sample excluded		17	0.24	[0.08; 0.39]	12	**.002**
**Subgroup analyses**						
Study design	Within	5	0.28	[0.05; 0.52]		.51
	Between	13	0.18	[− 0.009; 0.37]		
Cue type	Visual	6	0.21	[− 0.06; 0.48]		.95
	Auditory	6	0.20	[− 0.03; 0.43]		
	Label	6	0.26	[− 0.09; 0.62]		
Cue method	Single	12	0.14	[− 0.06; 0.36]		.41
	Double	5	0.35	[0.12;.58]		
	Mixed	1	0.34	[− 0.17; 0.85]		

#### Age of autobiographical memories (AMs)

The overall results showed a significant positive effect (*g* = 0.53, 95% CI [0.28; 0.77]), indicating that odor-evoked AMs are older or more likely to come from childhood than those evoked by other cues, and the heterogeneity is also low (*I*^*2*^ = 12%) (see Table 4)*.* No sensitivity analysis excluding clinical samples is reported for this outcome, as none of the studies contributing to this effect size included a clinical sample. However, note that this analysis is limited by the lack of consensus in the literature on how to measure the age of AMs and should therefore be interpreted with caution, given the small number of studies included in the analysis.

**Table 4:** Overall results and subgroup analyses for the age of autobiographical memories (AMs) (olfactory cue vs. other cue types)

		*k*	*g*	95% CI	*I*^*2*^	*p*
**Age of AMs**						
All studies		8	0.53	[0.28; 0.77]	12	** <.001**
**Subgroup analyses**						
Study design	Within	2	0.29	[− 0.04; 0.64]		.08
	Between	6	0.69	[0.39; 0.98]		
Cue type	Visual	2	0.55	[− 0.06; 1.17]		.99
	Auditory	2	0.52	[− 0.02; 1.07]		
	Label	4	0.55	[0.10; 1.00]		
Cue method	Single	6	0.57	[0.27; 0.87]		.53
	Double	2	0.37	[− 0.17; 0.92]		

#### Rarity

In terms of rarity, the overall results did not show a significant advantage of olfactory cues compared with other cue types (*p* =.10), and the level of heterogeneity was moderate (*I*^*2*^ = 56%). This suggests that odor-evoked AMs are not perceived overall as rarer than those evoked by other cues. However, after excluding the clinical samples, the effect became significant (*g* = 0.37, 95% CI [0.14; 0.59], *p* =.001). Unlike the other six outcomes, for which the sensitivity analysis removed at most a single contrast, rarity was the only outcome contributed by two separate clinical samples (Leiker et al., 2024; Lopis et al., 2021). Consequently, we analyzed each contribution individually. Excluding Leiker et al. (2024) alone left the effect non-significant (*g* = 0.24, 95% CI [− 0.07; 0.56], *p* =.13), whereas excluding Lopis et al. (2021) alone resulted in a significant effect favoring olfactory cues (*g* = 0.33, 95% CI [0.13; 0.54], *p* =.001) and reduced heterogeneity from *I*^*2*^ = 56% to 0%. This indicates that the difference between the overall and sensitivity analyses, as well as the level of heterogeneity, is specifically attributable to the Lopis et al. (2021) study (see Table 5).

**Table 5:** Overall results and subgroup analyses for rarity of evoked autobiographical memories (olfactory cue vs. other cue types)

		***k***	***g***	95% CI	***I***^***2***^	***p***
**Rarity**						
All studies		11	0.23	[− 0.05; 0.51]	56	.10
Clinical sample excluded		9	0.37	[0.14; 0.59]	0	**.001**
**Subgroup analyses**						
Study design	Within	1	0.19	[− 0.29; 0.67]		.86
	Between	10	0.24	[− 0.07; 0.56]		
Cue type	Visual	5	− 0.009	[− 0.43; 0.41]		.09
	Label	6	0.40	[0.15; 0.66]		
Cue method	Single	11	-	-		

#### Specificity and vividnes

In terms of specificity and vividness, the results did not show a significant effect of olfactory cues in contrast to other cue types: specificity (*p* =.30), vividness (*p* =.07). The effect remained non-significant after excluding the clinical samples. This means that odor-evoked AMs are not perceived as more detailed and vivid than AMs evoked by other cue types (see Table 6).

**Table 6:** Overall results and subgroup analyses for vividness and specificity of evoked autobiographical memories (olfactory cue vs. other cue types)

		*k*	*g*	95% CI	*I*^*2*^	*p*
**Vividness**						
All studies		18	0.14	[− 0.01; 0.31]	28	.07
Clinical sample excluded		17	0.12	[− 0.04; 0.29]	30	.15
**Subgroup analyses**						
Study design	Within	3	0.21	[− 0.03; 0.46]		.49
	Between	15	0.10	[− 0.10; 0.31]		
Cue type	Visual	8	0.27	[0.10; 0.43]		.16
	Auditory	4	− 0.005	[− 0.37; 0.36]		
	Label	6	− 0.06	[− 0.47; 0.33]		
Cue method	Single	9	0.11	[− 0.18; 0.41]		.48
	Double	8	0.12	[− 0.08; 0.33]		
	Mixed	1	0.45	[− 0.06; 0.96]		
**Specificity**						
All studies		12	0.07	[− 0.07; 0.22]	10	.30
Clinical sample excluded		11	0.06	[− 0.09; 0.22]	17	.43
**Subgroup analyses**						
Study design	Within	4	0.08	[− 0.13; 0.31]		.77
	Between	8	0.04	[− 0.19; 0.27]		
Cue type	Visual	5	0.13	[− 0.07; 0.33]		.39
	Auditory	5	0.06	[− 0.18; 0.30]		
	Label	2	− 0.31	[− 0.91; 0.29]		
Cue method	Single	4	0.17	[− 0.12; 0.47]		.07
	Double	7	− 0.005	[− 0.17; 0.16]		
	Mixed	1	0.60	[0.08; 1.12]		

#### Subgroup analyses

Subgroup analyses showed a significant moderating effect (Q(1) = 6.825, *p* =.009) of study design on *emotional intensity*. Studies that used a within-subject design reported greater advantage for olfactory cues over other cue types compared with between-subject studies (see Table 2). Regarding the other outcomes analyzed, study design was not a significant moderator (see Tables 2, 3, 4, 5, and 6).

Cue type compared with olfactory cues (i.e., auditory, visual, or label cues) showed a significant moderating effect (Q(2) = 9.128, *p* =.01) on the *pleasantness* of evoked AMs. There was a stronger effect in favor of olfactory cueing compared to label cueing, followed by visual and auditory cueing (see Table 2). No other moderating effects of cue type on other outcomes were found (see Tables 2, 3, 4, 5, and 6).

Finally, the cue method (i.e., single-cue, double-cue, and mixed) did not show any moderating effect on any of the outcomes analyzed (see Tables 2, 3, 4, 5 and 6)*.*

#### Meta-regression analyses

For sample size, the number of participants was introduced as a predictor for the effect size. The results showed that sample size was a significant moderator for emotional intensity (*slope* = − 0.004, 95% CI [− 0.007; − 0.001], *p* =.003), pleasantness (*slope* = − 0.005, 95% CI [− 0.011; − 0.00002], *p* =.04) and rarity (*slope* = − 0.007, 95% CI [− 0.010; − 0.003], *p* <.001). These results suggest that studies with larger samples reported a smaller effect of olfactory cues compared to other cue types for emotional intensity, pleasantness, and rarity of AMs (see Fig. 5). However, the association for rarity was no longer significant (*p* =.63) after excluding Lopis et al. (2021), whose removal also eliminated the heterogeneity observed for this outcome (see Main results). Therefore, this result should be interpreted with caution.

Additionally, the meta-regression analyses revealed a significant negative association between the effect size of evocativeness and the percentage of females (*slope* = − 0.018, 95% CI [− 0.032; − 0.004], *p* =.01), suggesting that an increase in the percentage of females is associated with a decrease in the advantage of olfactory cues over other cue types on evocativeness of AMs (see Fig. 6A).

Finally, publication year was a significant moderator for pleasantness (*slope* = − 0.019, 95% CI [− 0.035; − 0.004], *p* =.01). This result suggests that an increase in publication year corresponds to a decrease in the advantage of olfactory cues over other cue types on the pleasantness of AMs (see Fig. 6B).

No other meta-regression analysis was significant.

#### Publication bias

**Fig. 3:**
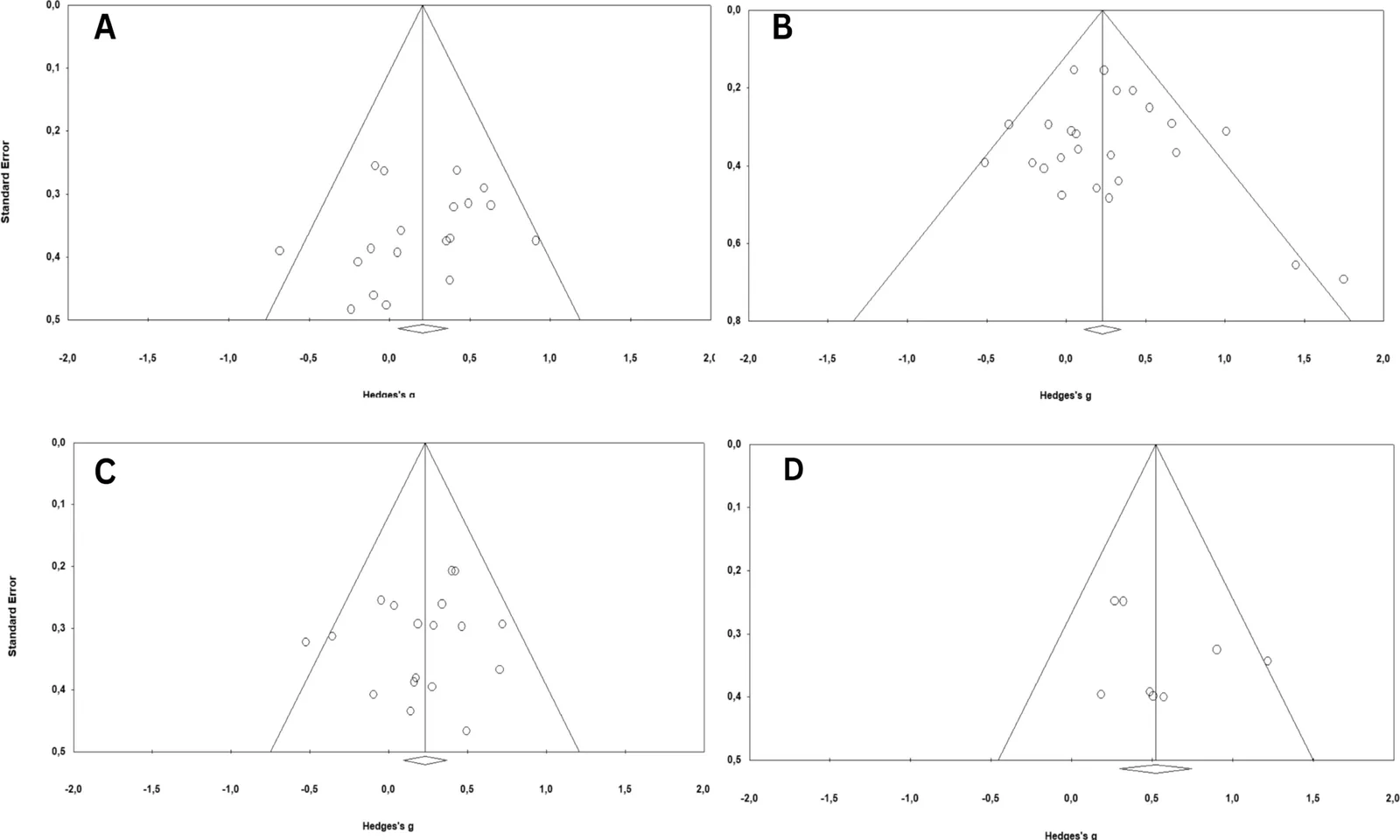
Funnel plots for olfactory cues vs. other cue types on pleasantness (**A**), emotional intensity (**B**), evocativeness (**C**), and age of autobiographical memories (**D**)

**Fig. 4:**
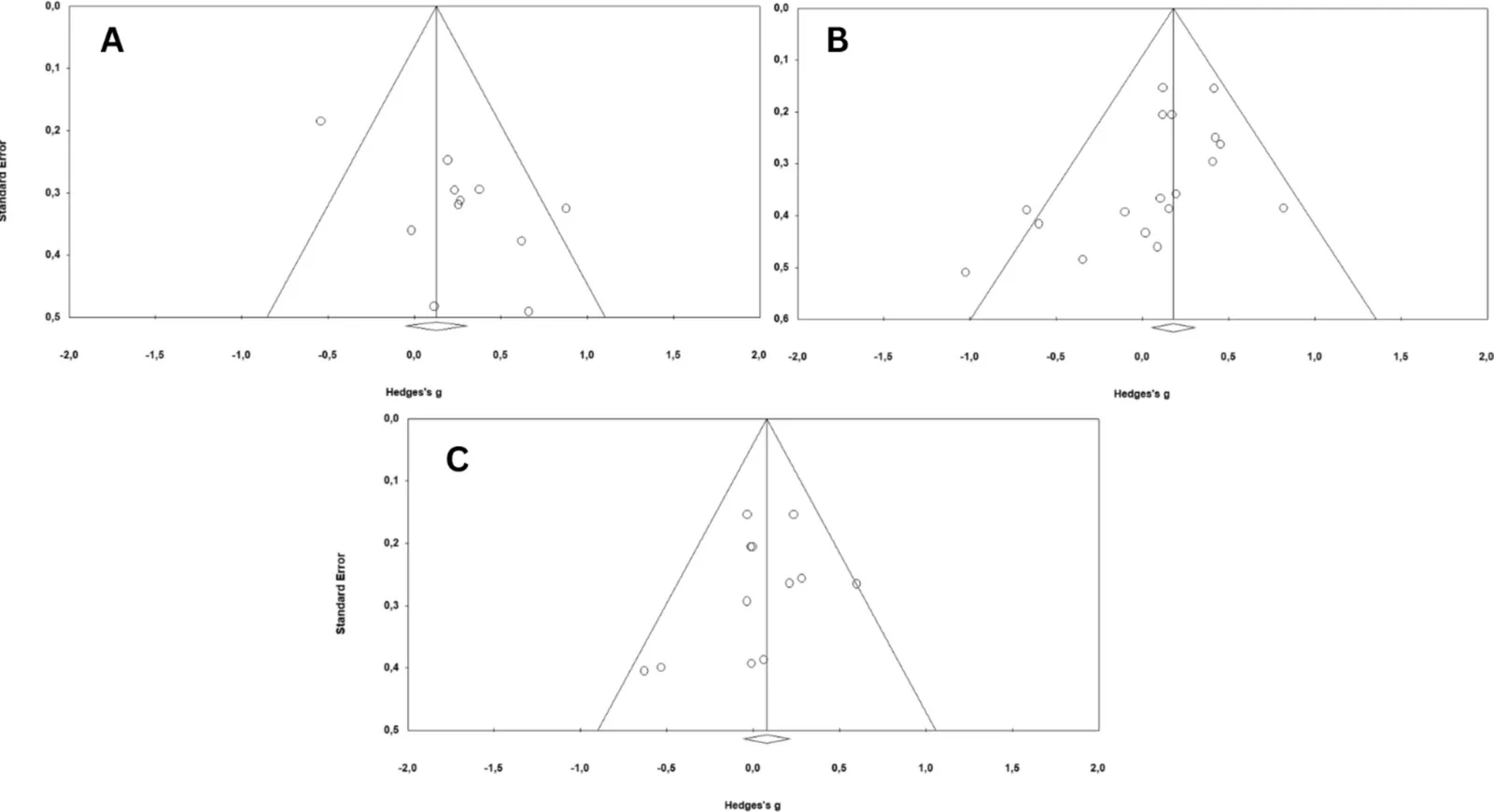
Funnel plots for olfactory cues vs. other cue types on rarity (**A**), vividness (**B**), and specificity (**C**)

We assessed publication bias for the pooled effect sizes of each of the seven outcomes. Visual inspection of the funnel plots showed that effect sizes were evenly distributed around the pooled estimate for six of the seven outcomes (see Figs. 3 and 4). For rarity, a single contrast fell outside the funnel at high precision. Egger’s test was non-significant for five of the seven outcomes (*p* >.05); the two exceptions were vividness (*intercept* = − 1.411, 95% CI [− 2.819; − 0.003], *p* =.04) and rarity (*intercept* = 3.785, 95% CI [0.934; 6.636], *p* =.01). However, for rarity, Egger’s test became non-significant (*p* =.55) after excluding the Lopis et al. (2021) study. Finally, Duval and Tweedie’s trim-and-fill procedure estimated no missing studies for six of the seven outcomes. For rarity, five missing studies were estimated, a number that fell to zero after exclusion of the Lopis et al. (2021) study. Given the substantial heterogeneity for this outcome (*I*^*2*^ = 56%) and the disproportionate influence of a single study (see Main results), this estimate should be interpreted with caution, as trim-and-fill performance is known to be unreliable under substantial between-study heterogeneity and can suggest publication bias even in its absence (Peters et al., 2007).

## Discussion

The present study is the first meta-analysis to investigate the advantage of olfactory cues compared to other cue types in evoking more emotionally intense, pleasant, evocative, vivid, specific, rare, and older AMs. In addition, a comprehensive list of moderators was analyzed; risk of bias and publication bias were also assessed. The main findings are discussed below.

The results showed that odor-evoked AMs are perceived as more pleasant and emotionally intense than AMs evoked by other cues. This result confirms the Proustian idea that odor-evoked AMs are experienced as more emotionally intense than AMs evoked by other cues (Chu & Downes, 2000; Hackländer et al., 2019), while the significant effect on pleasantness is consistent with previous research (Hackländer et al., 2019), and with the therapeutic relevance of olfactory cueing (Herz, 2016). These effects may partly reflect the intrinsic nature of olfaction itself. Some studies show a convergent relationship between odor and affective processes, in which the neuroanatomy of the olfactory system and the circuits underlying emotion are closely linked (see Kontaris et al., 2020, for a review), potentially influencing what or how AMs are evoked.

Additionally, the nostalgia hypothesis (Hackländer et al., 2019) may explain these findings. Some research suggests that olfactory cues are associated with experiencing nostalgia (Reid et al., 2015), due to their characteristic of evoking AMs more likely to come from childhood (Larsson et al., 2014), and with age, childhood tends to be reappraised as more emotionally intense and positive than it was at encoding (Levine & Safer, 2002). It should be noted, however, that direct empirical support for this hypothesis remains limited (Janssen & Murre, 2008).

Moreover, most studies used pleasant odors, which could have introduced a recall bias. For example, Bogenschütz et al. (2022) argued that emotionality typically found in odor-evoked AM research may reflect the influence of the odor on the retrieval experience rather than intrinsic properties of the odor-evoked AMs themselves. Consequently, the observed effects on pleasantness and emotional intensity may partly reflect the characteristics of the stimuli selected rather than the mnemonic advantage specific to the olfactory modality.

Beyond the selected odors, another aspect to consider is the wide variability in odor hedonic perception. How an odor is perceived varies considerably across individuals, shaped by culture, physiological state, identification ability, and prior experience (Bontempi et al., 2024). Therefore, the advantage of olfactory cues observed here might reflect, at least in part, the extent to which the odors were consistently perceived by each participant, rather than a stable characteristic of olfactory cueing itself. Future studies are needed to directly evaluate the influence of olfactory hedonic perception on the emotional dimensions of odor-evoked AMs.

Similar to previous findings (Hackländer et al., 2019), our results showed that odor-evoked AMs were characterized by a stronger reliving of AMs than those evoked by other cues. These findings are consistent with the Proustian hypothesis regarding the evocative quality of the odor-evoked AMs (Larsson & Willander, 2009; Larsson et al., 2014).

Moreover, in agreement with the characterization of the Proust phenomenon, our results showed that odor-evoked AMs tend to originate earlier in life than those evoked by other cues. Note that this analysis is limited by the lack of consensus in the literature on how to measure the age of AMs. Therefore, the variety of analyses regarding the age of AMs did not allow us to compute all effect sizes in a global analysis, as there are studies that have used the decades of life paradigm to test the age of odor-evoked AMs (Willander & Larsson, 2006, 2007; Willander et al., 2015). Consequently, this result should be interpreted with caution, given the small number of studies included in the analysis.

Interestingly, we found no evidence of an olfactory advantage over other cue types for rarity, vividness, or specificity. In other words, odor-evoked AMs were not perceived as more vivid, rare, or detailed than AMs evoked by other cue types. These findings contrast with prior evidence regarding the vivid, rare, and specific quality of odor-evoked AMs (Hackländer et al., 2019; Larsson et al., 2014). A potential reason for these conflicting results is that some narrative reviews based their conclusions on studies that used lab-created artificial episodes (Larsson & Willander, 2009; Larsson et al., 2014) rather than personal life events recalled. In addition, the analyses of the previous review (Hackländer et al., 2019) were, in some cases, based on preferential data extraction. For instance, the vividness analysis was restricted to childhood odor data, which may have contributed to an overestimation of the effect for this outcome.

However, the evidence for rarity was less clear. Although the overall effect was not significant, this outcome showed considerable heterogeneity, attributable specifically to one study (see Results section). Excluding this study (Lopis et al., 2021) both resolved the heterogeneity and rendered the pooled effect significant. Given this divergence between the overall and sensitivity analyses, as well as the asymmetry of the funnel plot attributable to the same contrast (see Publication bias), the overall null effect for rarity should be interpreted with more caution than that observed for other outcomes, as the available evidence does not conclusively rule out an olfactory advantage.

### Moderator effects

Moderating effects are discussed below, considering subgroup and meta-regression analyses.

### Subgroup analyses

Firstly, study design had a significant effect on the emotional intensity of AMs. Specifically, within-subject studies reported a greater advantage of olfactory cues over other cue types than between-subject studies. This pattern is consistent with the broader methodological literature (Charness et al., 2012), which shows that within-subject designs, where each participant is exposed to all conditions, produce greater statistical power and reduce inter-individual variability (Strale, 2024). This may be particularly relevant in our analyses, given the wide variability in olfactory perception (Bontempi et al., 2024).

Secondly, cue type had a significant effect on AMs’ pleasantness. The results showed that olfactory cues evoked more pleasant AMs than label cues, followed by visual and auditory cues. These findings are noteworthy, as the subgroup analyses provide the first meta-analytic evidence that the advantage of olfactory cues on the pleasantness of AMs varies depending on the cue type compared. A possible explanation for this moderating effect may be related to the semantic processing hypothesis. Odors are more difficult to identify than other sensory cues, partly due to the inefficient activation of lexical representations (Olofsson & Gottfried, 2015). Furthermore, as Olofsson and Gottfried (2015) have argued, nonverbal auditory cues may share some of these semantic limitations, an idea consistent with Karlsson et al. (2013), who found that while visually evoked AMs differed significantly in their semantic representations from both olfactory and auditory cues, the difference between olfactory and auditory cues was only marginal. Consequently, the advantage of olfactory cueing on the pleasantness of evoked AMs may be more pronounced when compared to cueing modalities that rely predominantly on semantic processing, such as label cues, and attenuated relative to modalities that share similar semantic constraints, such as auditory cues. However, experimental studies that directly manipulate the semantic accessibility of different cue types are needed to test these assumptions.

Nevertheless, some overall effects were not consistently moderated across subgroups. Some loss of power is expected in this context due to the smaller number of contrasts in certain subgroups. Furthermore, a possible reduction in statistical power, linked to the sample-splitting approach used to address dependence among contrasts, cannot be ruled out.

Finally, subgroup analyses on the cue method were not significant. Therefore, heterogeneity could not be explained by differences in cue methods (single, double, or mixed). A likely reason is that our meta-analysis was underpowered to identify small effects in some subgroups.

### Meta-regression analyses

**Fig. 5:**
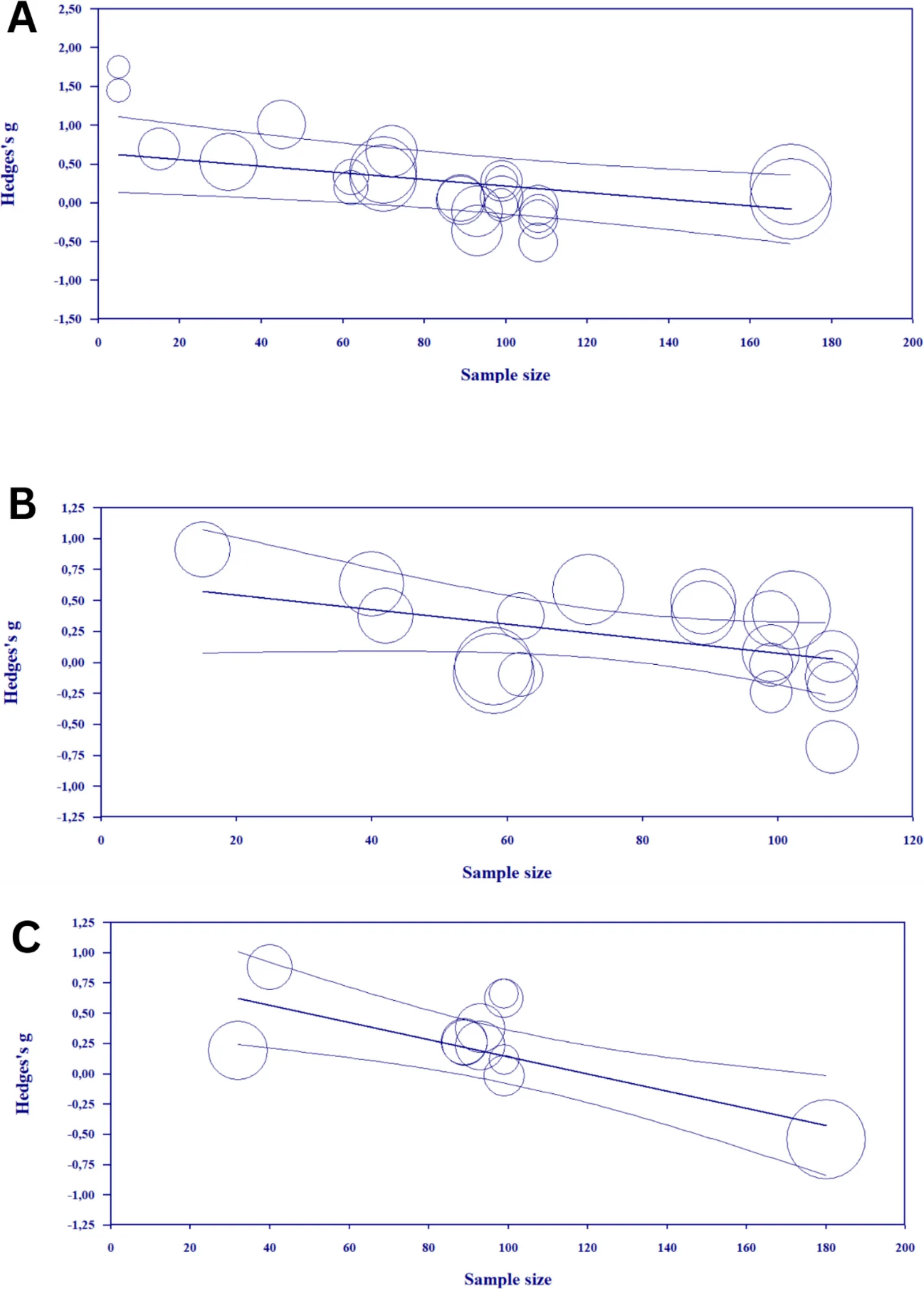
Slope analysis depicting effect size depending on sample size for emotional intensity (**A**), pleasantness (**B**), and rarity (**C**)

In terms of meta-regressions, the sample size emerged as a significant moderator for emotional intensity, pleasantness, and rarity of AMs (Fig. 5). Specifically, the relative advantage of olfactory cues over other cue types decreased as sample size increased, indicating that studies with larger samples reported smaller effect sizes. This is a common pattern in psychological research, in which studies with small samples often produce larger effect sizes than those using large samples (Kühberger et al., 2014). However, for rarity, this association was no longer significant after excluding Lopis et al. (2021), the largest study contributing to this outcome, and should therefore be interpreted with caution.

**Fig. 6:**
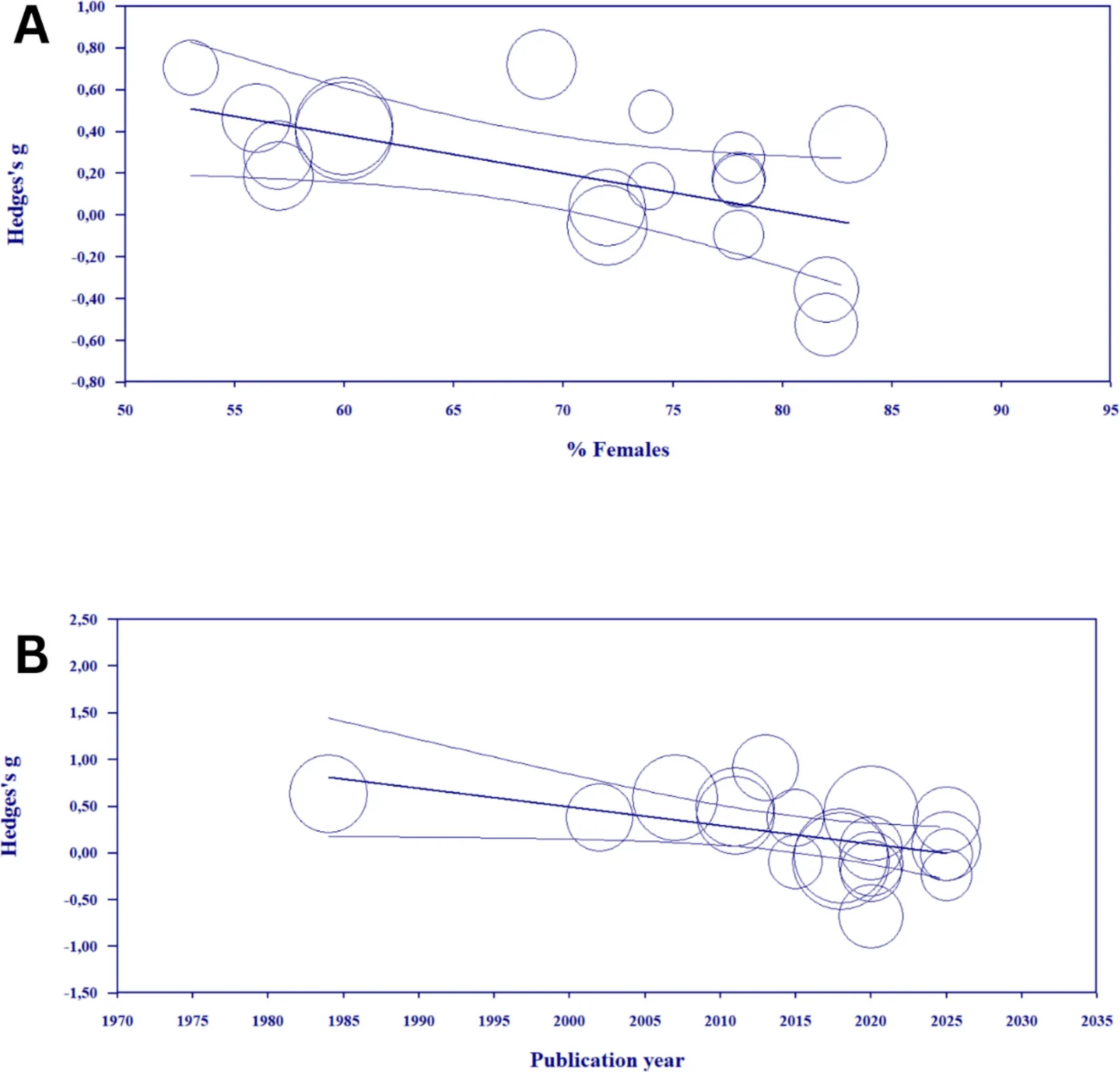
Slope analysis depicting effect size depending on the percentage of females for evocativeness (**A**), and publication year for pleasantness (**B**)

Additionally, meta-regression analyses showed that the percentage of females moderated AMs’ evocativeness (Fig. 6A). The results showed that an increase in the percentage of females was associated with a decrease in the effect size. Gender differences in AMs are a widely studied phenomenon (Davis, 1999; Fivush, 2011; Seidlitz & Diener, 1998; Zucco et al., 2012), with evidence suggesting that women tend to recall AMs in greater detail and elaboration than men (e.g., Fivush, 2011). Therefore, a possible explanation is that, in samples with more women, AMs may be perceived as highly evocative regardless of the sensory cue used. Consequently, the relative advantage of olfactory cues over other cue types may be reduced in samples with a higher proportion of females.

Finally, publication year was a significant moderator for pleasantness (Fig. 6B). The results showed that later studies were associated with smaller effect sizes than earlier ones. This trend is typical in experimental research, where first publication bias favors studies that confirm hypotheses, and then negative findings gradually emerge (e.g., Podina & Fodor, 2018).

No other meta-regression analysis was significant. This is likely attributable, in part, to the low heterogeneity observed across studies, which indicates limited variability in effect sizes available for moderators to explain. Additionally, the relatively small number of studies included in the analyses may have limited statistical power to detect moderating effects.

### Limitations and implications for future research

This meta-analysis has several limitations that need to be addressed. Firstly, the number of studies included in this analysis is relatively small. Although there were 18 studies with contrasts ranging from eight to 24 depending on the outcome analyzed, this meta-analysis may not have had the statistical power to detect certain effect sizes. In this regard, future studies with larger samples would be valuable for establishing more precise estimates of the olfactory cueing effect in contrast to other cueing modalities, especially for emotional intensity, pleasantness, and rarity of AMs, where the negative association with sample size suggests a potential amplification of effects in smaller studies. In line with this, the non-significant effect observed for rarity should be interpreted with caution, as it was sensitive to the exclusion of the study with the largest sample.

Secondly, the number of comparisons in some subgroup analyses was low. For example, there were only two comparisons between olfactory versus auditory cues and between olfactory versus visual cues on the age of AMs. Thus, these findings need to be interpreted with caution.

In line with this, the included studies reported only comparisons between olfactory, visual, auditory, and label cueing. Therefore, these findings cannot be extrapolated to the full range of cueing modalities, given the lack of comparisons between olfactory and tactile or gustatory cues. Future research should consider these points to better understand the Proust phenomenon. Otherwise, there is a risk of developing conceptualizations of the phenomenon in the absence of experimental data. Moreover, future research should also investigate other characteristics associated with odor-evoked AMs, such as the feeling of surprise (Hackländer et al., 2019). Extending comparisons to these less-studied modalities and features of AMs would allow a more comprehensive analysis of the Proust phenomenon.

Thirdly, because each of the seven AM outcomes was tested in a separate model, the risk of a Type I error cannot be excluded. However, these outcomes reflect conceptually distinct dimensions of odor-evoked AMs, which have been treated as separate constructs in prior work (e.g., Chu & Downes, 2000; Hackländer et al., 2019), justifying separate analyses. A related consideration concerns the sample-splitting approach used for contrasts sharing the same olfactory sample within a study. We adopted this approach because the number of studies contributing multiple contrasts per outcome was limited, making sample-splitting a stable solution under these conditions. Future research could apply corrections for multiple comparisons across outcomes and adopt approaches such as robust variance estimation (RVE; Sidik & Jonkman, 2006), which may become better suited to modeling contrast dependency directly as more studies emerge.

Fourthly, the variety of analyses regarding the age of AMs did not allow us to compute all effect sizes in a global analysis, as there are studies that have used the decades-of-life paradigm to test the age of odor-evoked AMs (Willander & Larsson, 2006, 2007; Willander et al., 2015). Thus, the analysis of the age of AMs should be interpreted as an exploratory analysis rather than a primary one.

Additionally, given that odor hedonic perception varies across individuals (Bontempi et al., 2024), future research should more directly assess this variability when evaluating olfactory cues relative to other cue types. For example, odor concentration and intensity were not systematically reported in the included studies, despite evidence that these properties substantially shape hedonic and emotional responses to odors (Bontempi et al., 2024). At the individual level, direct measures of olfactory acuity or identification ability (e.g., standardized psychophysical tests) were rarely reported, even though such measures could clarify whether the advantage of olfactory cueing depends on participants’ baseline olfactory function rather than on the modality itself.

Finally, although this meta-analysis focused on autobiographical memory, i.e., the paradigm involving personal life events, parallels can be drawn with episodic memory research, where sensory modalities are often compared more systematically. In line with this, integrating insights from both approaches could help determine whether the distinctiveness of olfactory cueing is specific to personally significant memories or reflects broader mechanisms of sensory-dependent episodic retrieval.

### Implications

The implications of the Proust phenomenon extend beyond the theory regarding how autobiographical memory works under conditions of sensory cueing. In clinical practice, interest in using sensory cues, especially for therapeutic purposes, has increased considerably. There are studies that have tried to use odors to improve participants’ narrative storytelling (Choi et al., 2024; Leret & Visch, 2017), while other studies have shown improvement in memory, attention, and language through odor-based exposure among people with dementia (e.g., Cha et al., 2022). In addition, studies show that odor exposure can help Alzheimer’s patients retrieve AMs (Arroyo-Anlló et al., 2020; El Haj, 2022).

## Conclusions

The present study is the first meta-analysis to address the Proust phenomenon by examining the advantage of olfactory cueing over other cueing modalities in retrieving AMs. Seven key characteristics of evoked AMs were analyzed: *emotional intensity*, *pleasantness, evocativeness*, *rarity, age of AMs, vividness,* and *specificity*. Olfactory cues showed an advantage over other cue types for emotional intensity, pleasantness, evocativeness, and age of AMs. However, this advantage could not be found for rarity, vividness, and specificity. Additionally, this meta-analysis identified several moderators of the variability in effect sizes. Specifically, sample size was a significant moderator for emotional intensity, pleasantness, and rarity, indicating that studies involving larger samples were associated with smaller effects. Similarly, the percentage of females moderated evocativeness, and the publication year moderated pleasantness, both showing negative relationships with the effect size. Moreover, subgroup analyses indicated that study design moderated emotional intensity, with within-subject studies producing greater effects favoring olfactory cueing over other cueing modalities than between-subject designs. Finally, the strongest advantage was observed when olfactory was compared to label cues, followed by visual and auditory cues on the pleasantness of evoked AMs. Future research is needed to further clarify the conditions under which olfactory cueing has a more pronounced effect on evoked AMs relative to other cueing modalities.

## Data Availability

The datasets generated and/or analyzed during the current study are available from the corresponding author on reasonable request. Data coded for the moderator analyses are partly presented in the study characteristics table.

## Code availability

All procedures and software used are described in the Methods section.
